# Oxidation Behavior of Aluminide Coatings on Cobalt-Based Superalloys by a Vapor Phase Aluminizing Process

**DOI:** 10.3390/ma17235897

**Published:** 2024-12-02

**Authors:** Kuo Ma, Cheng Xie, Yidi Li, Biaobiao Yang, Yuanyuan Jin, Hui Wang, Ziming Zeng, Yunping Li, Xianjue Ye

**Affiliations:** 1 State Key Laboratory of Powder Metallurgy, Central South University, Changsha 410083, China; makuo@csu.edu.cn (K.M.); youxianboy@163.com (C.X.); liiidi@csu.edu.cn (Y.L.); jinyuanyuan83@csu.edu.cn (Y.J.); wanghuiii@csu.edu.cn (H.W.); zndxzzm@csu.edu.cn (Z.Z.); 2 AECC South Industry Company Limited, Zhuzhou 412002, China; 3 IMDEA Materials Institute, C/Eric Kandel 2, Getafe, 28906 Madrid, Spain; biaobiao.yang@imdea.org; 4 Department of Materials Science, Polytechnic University of Madrid/Universidad Politécnica de Madrid, E.T.S. de Ingenieros de Caminos, 28040 Madrid, Spain; 5 School of Materials Science and Engineering, Zhejiang University, Hangzhou 310058, China

**Keywords:** aluminide coating, cobalt-based superalloy, vapor phase aluminizing, oxidation

## Abstract

In this work, the oxidation behavior of an aluminide coating at 900, 1000, and 1100 °C was investigated. The aluminide coating was prepared on a cobalt-based superalloy using a vapor phase aluminizing process, which is composed of a β-(Co,Ni)Al phase outer layer and a Cr-rich phase diffusion layer. The experimental results showed that the oxidation of the coating at 900–1100 °C all obey the parabolic law. The oxidation rate constants of the coating were between 2.19 × 10^−7^ and 47.56 × 10^−7^ mg^2^·cm^−4^·s^−1^. The coating produced metastable θ-Al_2_O_3_ at 900 °C and stable α-Al_2_O_3_ at 1000 and 1100 °C. As the oxidation temperature increases, the formation of Al_2_O_3_ is promoted, consuming large amount of Al in the coating, resulting in the transformation from β-(Co,Ni)Al phase to α-(Co,Ni,Cr) phase. And the decrease in the β phase in the coating led to the dissolution of the diffusion layer.

## 1. Introduction

Cobalt-based superalloys exhibit excellent comprehensive mechanical properties at high temperatures, which makes them widely used in the manufacture of gas turbine engine blades for aerospace and marine applications [1,2,3]. To enhance the thrust-to-weight ratio and thermal efficiency of these engines, it is imperative to elevate the operating temperature of these engines [4]. Despite the continuous development of cobalt-based superalloys, it is difficult to work properly at such high temperatures due to the lack of oxidation resistance. Hence, the application of high-temperature protective coatings on cobalt-based superalloys is very essential to improve the oxidation and corrosion resistance of cobalt-based superalloys in high-temperature environments [5]. Aluminide coatings stand out as the most prevalent, cost-effective, and straightforward method for high-temperature protection, including pack cementation, slurry cementation, hot dip aluminizing, chemical vapor deposition (CVD), and vapor phase aluminizing [6,7,8,9]. The vapor phase aluminizing deposits aluminum onto the surface of the substrate in a vapor phase by a chemical vapor phase reaction. In this process, the substrate is placed above and away from the aluminizing agent [10]. The vapor phase aluminizing process has the advantages of good coating quality, uniform coating, and being not easy to block the air holes of hollow turbine blades [11].

The fabrication of aluminide coatings on cobalt-based superalloys presents a greater challenge compared to their nickel-based counterparts. This is primarily due to the fact that the formation of CoAl is inherently more difficult than that of NiAl [12]. There have been many studies of aluminide coatings on nickel-based superalloys, including the exploration of aluminizing process [13,14], the modification of aluminide coatings [15,16], and the research of coatings properties [6,17]. In contrast to the extensive research of aluminide coatings on nickel-based superalloys, there has been some research of aluminide coatings on cobalt-based superalloys. Lee and Kuo [18] prepared β-CoAl coatings on an AMS 5608 alloy via a pack cementation process and found that the diffusion of Al into the substrate and the cracks and holes in the diffusion zone were the main reasons for the failure of the coating. Wang and Sayre [19] reported that β-CoAl coatings and Pt-modified aluminide coatings on Haynes 188 and WI-52 alloys with a vapor phase aluminizing process and found that the substrate composition affects the formation of the coatings. Despite these studies, there are very few studies on the preparation of aluminide coatings on cobalt-based superalloys by vapor phase aluminizing process, so there is a need for more research.

Some research has been carried out on the oxidation resistance of aluminide coatings on cobalt-based superalloys. Liu et al. [20,21] found that the isothermal oxidation of aluminide coatings of DZ40M alloy at 900–1100 °C can be divided into three stages: initial oxidation, stable oxidation, and accelerated oxidation. It has also been found that Ti accelerates the failure of the coating during the oxidation. Feng et al. [22,23] studied the oxidation behavior of Si-modified aluminide coatings and Dy, Si-modified aluminide coatings on a new type of γ′-strengthened cobalt-based superalloy. The results showed that both Si and Dy could improve the oxidation resistance of the coatings. However, the oxidation behavior of aluminide coatings on cobalt-based superalloys has not been thoroughly investigated, and further studies are needed.

Therefore, in this study, aluminide coatings were prepared on cobalt-based superalloys via a vapor phase aluminizing process, and isothermal temperature oxidation experiments were carried out at 900, 1000, and 1100 °C to investigate the oxidation behavior of the coating at different temperatures.

## 2. Experiment

The material for this study was a cast cobalt-based superalloy K6509, which was fabricated by the Beijing Institute of Aeronautical Materials (Beijing, China) [24], and the composition is tabulated in Table 1. The alloy ingot was cut into 15 mm × 10 mm × 4 mm samples by an electric discharge machine. Then, a round hole is drilled through the samples using a drilling machine to facilitate the hanging of the samples during the preparation of the coating. The samples were ground with 80#, 240#, 600#, and 1000# sandpaper in turn followed by cleaning in alcohol solution.

**Figure 1:**
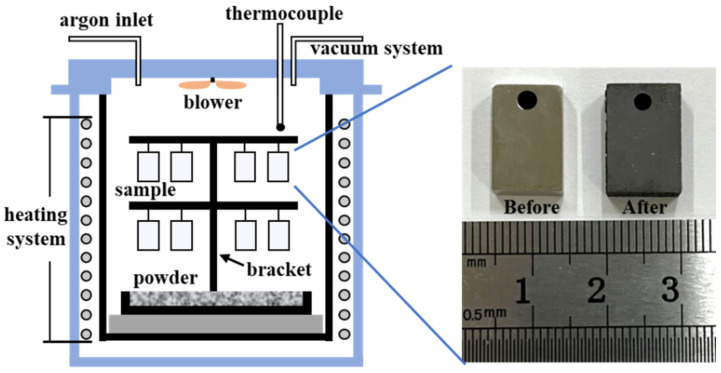
Schematic drawing of vapor phase aluminizing furnace.

The Al powder (2N, Fushel, Changsha, China), Al_2_O_3_ powder (AR, Sinopharm Chemical Reagent Co., Ltd., Shanghai, China), and NH_4_Cl powder (AR, XiLong Scientific, Shantou, China) were accurately weighed in a mass ratio of 60:39:1. The ratio of the three powders was based on previous studies [19,20,22], and an initial estimation was made for the mass ratio of the three powders, which was then further refined through experiments to ensure that the desired coating quality was achieved. The three powders were put into a V-mixer (VH2, Tianhe Machinery, Shanghai, China) and mixed for 2 h to make them homogeneous to obtain the aluminizing agent. The schematic drawing of the vapor phase aluminizing furnace (VPAF1, CSU, Changsha, China) is shown in Figure 1. The samples were hung on a birdcage-type bracket by nickel wires, and the aluminizing agent was placed on the base of the bracket. Then, the furnace was pumped to vacuum and then filled with argon gas, and the operation was repeated three times to prevent the samples and the aluminizing agent from oxidation. Finally, the furnace was heated to 980 °C with a heating rate of 5 °C/min and held for 8 h. After the furnace cooled naturally to room temperature, the samples were taken out and cleaned with an alcohol solution.

The oxidation experiments were carried out at 900, 1000, and 1100 °C for 100 h under atmospheric conditions to investigate the oxidation behavior of aluminide coatings at different temperatures. Before the oxidation experiments, the alumina crucible used for the experiments was kept at 1200 °C for 12 h to decrease the experimental errors due to changes in crucible mass. At each temperature, three parallel samples were placed in three crucibles, and the initial mass of the samples and crucibles were accurately weighed using an electronic balance (ATY124, Shimadzu, Kyoto, Japan) with an accuracy of 10^−4^ g. The crucibles containing the samples were placed in a muffle furnace for isothermal oxidation. The crucibles were removed from the furnace at specified time points (1, 3, 5, 8, 12, 24, 48, 72, and 100 h), cooled for 30 min, and then accurately weighed the mass of the samples and crucibles.

The microstructure and elemental distribution of the coating before and after oxidation were analyzed using a scanning electron microscope (SEM, Quanta FEG 650, FEI, Hillsboro, OR, USA) with an energy dispersive spectroscopy (EDS). The phases of the coating before and after oxidation was analyzed via X-ray diffraction (XRD, Advance D8, Bruker, Fällanden, Switzerland). The Cu Kα (0.157 nm) radiation source used was scanned in the range of 20–90° with a scanning speed of 10°/min. Surface height profiles of the coating before and after oxidation were obtained using laser spectroscopy confocal microscope (KC-X1000, Kathmatic, Nanjing, China).

## 3. Result

### 3.1. The Microstructure of the Substrate and Coating Before Oxidation

**Figure 2:**
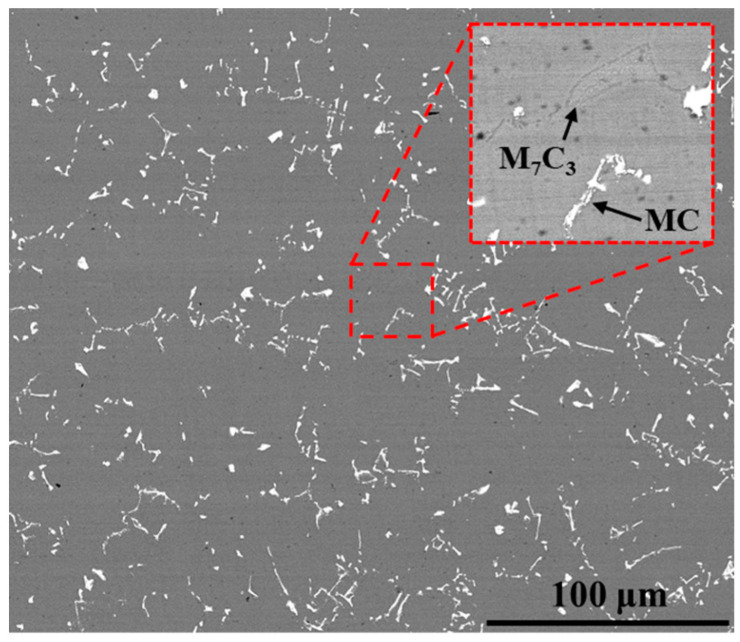
BSE image of the Co-base substrate K6509 microstructure.

The back scatter electron (BSE) image of the substrate is shown in Figure 2. K6509 is composed of solid solution matrix and a large number of carbides. The carbides include MC and M_7_C_3_, of which MC is Ta-rich carbide in the form of skeleton and M_7_C_3_ is Cr-rich carbide in the form of block [24]. The two carbides in Figure 2 were distinguished by EDS point spectrum analysis. The aluminizing temperature is lower than the melting temperature of the alloy, so it will not affect the substrate.

**Figure 3:**
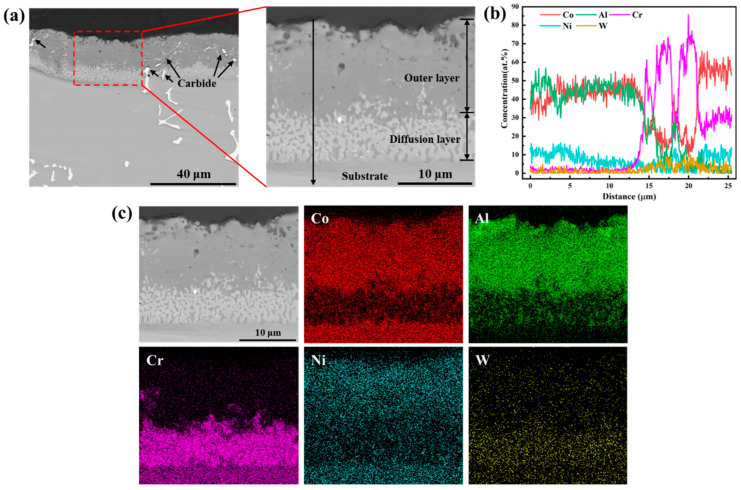
(**a**) Cross-sectional BSE images, (**b**) corresponding EDS line scanning results, and (**c**) elemental distribution maps of the coating.

Figure 3a shows the BSE images of the cross-section of the aluminide coating before oxidation. It can be seen that the prepared coating is relatively uniform with an outer layer and a diffusion layer, of which the total thickness is about 20 μm. The outer layer is characterized by a gray layer with some pores near the surface. And carbides can be observed in the outer layer. The diffusion layer between the outer layer and the substrate is half the width of the outer layer, in which some gray precipitates can be observed. The element distributions along the cross-section of the coating were characterized by the EDS line scanning and EDS map scanning, of which the results are shown in Figure 3b and Figure 3c, respectively. Combining the results in Figure 3b,c, it can be inferred that Co, Ni, and Al are mainly concentrated in the outer layer of the coating, while the diffuse layer is composed of a continuous precipitate phase rich in Cr. This is similar to Lee and Kuo’s study [17]. The chemical composition of the Cr-rich precipitate phase was analyzed by EDS point spectrum, which is 60.2Cr-22.4Co-7.2Al-5.1Ni-5.1W (at.%).

**Figure 4:**
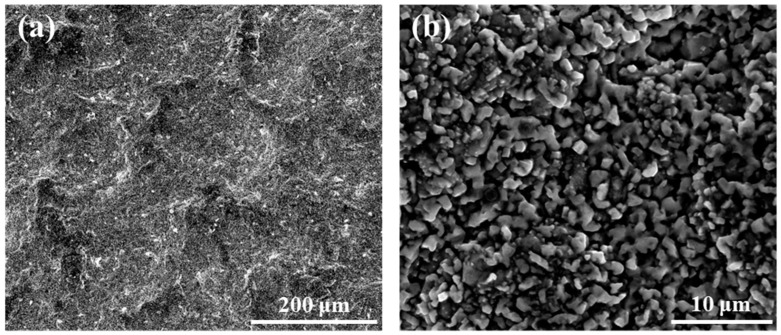
(**a**) Low-magnification and (**b**) high-magnification surface SE images of the coating.

The secondary electron (SE) images of surface morphologies of the coating are shown in Figure 4. The surface morphologies of the coating are relatively rough, which can be seen clearly in the low-magnification image (Figure 4a). The high-magnification image of the surface is shown in Figure 4b, in which the particles on the coating can be observed. The size of the particles exhibits heterogeneity, containing coarse particles and fine particles. In addition, many pores can be observed on the coating surface. The composition of the coating was obtained by EDS analysis as 44.5Co-39.0Al-14.0Ni-2.5Cr (at.%). 

**Figure 5:**
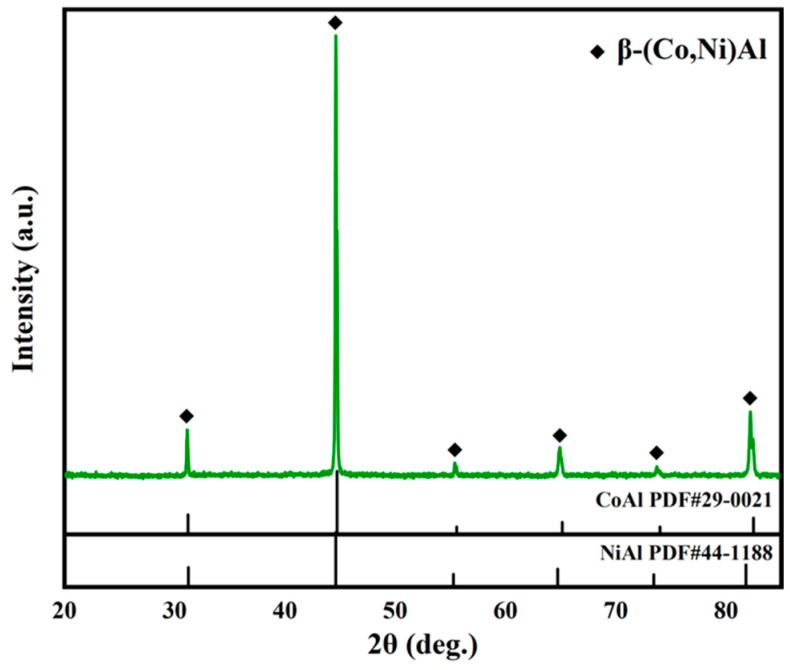
The XRD pattern of the coating.

Figure 5 exhibits the XRD pattern of the coating before oxidation. The coating is mainly composed of β-(Co,Ni)Al (abbreviated as β in the following paragraph), which is consistent with the elemental distributions of the coating cross-section shown in Figure 3c.

### 3.2. Oxidation Kinetic Curves

**Figure 6:**
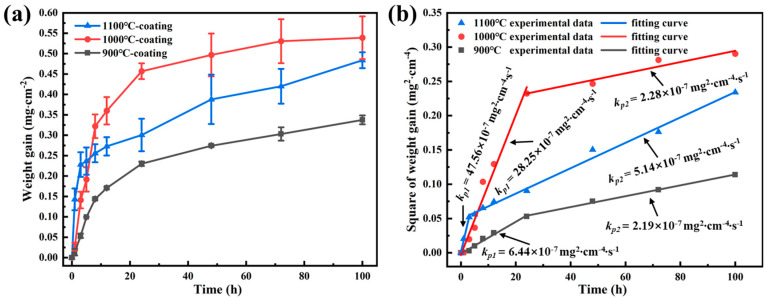
(**a**) Mass gain and (**b**) square of mass gain versus time of the coating at different temperatures for 100 h.

Figure 6a shows the mass gain of the coating as a function of oxidation time at 900–1100 °C. The mass gain is defined as the difference between the mass of the sample at each specific oxidation time point and the initial mass. It can be seen that the oxidation mass gain curves of the coatings at different temperatures are all nearly parabolic [25]. The mass gain of the coating at 900 °C is the lowest among three temperatures throughout the whole 100 h. In the aspect of mass gain at 1000 and 1100 °C, the oxidation mass gain of the coating at 1100 °C is larger than that at 1000 °C before 5 h. However, the mass gain rate at 1100 °C has slowed down significantly after 5 h, resulting in s lower mass gain compared to that at 1000 °C.

The mass gain curves of the coating at different temperatures all show a parabolic law, and their oxidation mass gain and oxidation time can be expressed as follows [26]:

(1) $$∆M^{2}=K_{P}\cdot t$$

∆M denotes the mass gain per unit area of the coating at a specified time point, $K_{P}$ is the oxidation rate constant, and t is the oxidation time. It has been shown that the curve of the mass gain squared for aluminide coatings can be divided into two stages, the rapid oxidation stage and the stable oxidation stage [27]. By linearly fitting $∆M^{2}$ and t, the $K_{P}$ of the coating at different temperatures can be obtained, as shown in Figure 6b. The $K_{P}$ of the coating for the rapid oxidation stage during oxidation at 900, 1000, and 1100 °C was 6.44 × 10^−7^, 28.25 × 10^−7^, and 47.56 × 10^−7^ mg^2^·cm^−4^·s^−1^, respectively, and then decreased to 2.19 × 10^−7^, 2.28 × 10^−7^, and 5.14 × 10^−7^ mg^2^·cm^−4^·s^−1^. $K_{P}$ increased with increasing temperatures in both stages.

**Figure 7:**
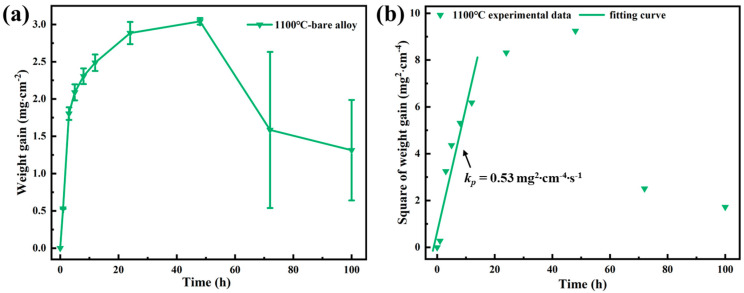
(**a**) Mass gain and (**b**) square of mass gain versus time of the bare alloy at 1100 °C for 100 h.

In order to compare the oxidation resistance of the coating with K6509 substrates, isothermal oxidation experiments at 1100 °C were also performed on bare alloys. Figure 7a shows the mass gain curve of the bare alloy oxidized at 1100 °C. The oxidation mass gain of the bare alloy at 1100 °C conforms to a parabolic law before 48 h, while the mass gain decreases significantly after 48 h. Since the samples and crucibles were weighed together, oxide scales spallation does not cause the mass gain to decrease. Therefore, the decrease in the weight gain may be ascribed to the production of volatile oxides during oxidation [28,29]. The oxidation of the bare alloy is not the focus of this study, so it will not be investigated in depth. In order to accurately evaluate the $K_{P}$ of the bare alloy at 1100 °C, the mass gain data before oxidation for 12 h were used to fit $∆M^{2}$ and t. The results are shown in Figure 7b. The $K_{P}$ of the bara alloy at 1100 °C was 0.53 mg^2^·cm^−4^·s^−1^. By comparing the oxidation of the coating with that of the bare alloy, it can be seen that the oxidation mass gain of the coating is much smaller than that of the bare alloy, indicating the excellent oxidation resistance of the coating in this study.

### 3.3. Oxidation Products at Different Temperatures

**Figure 8:**
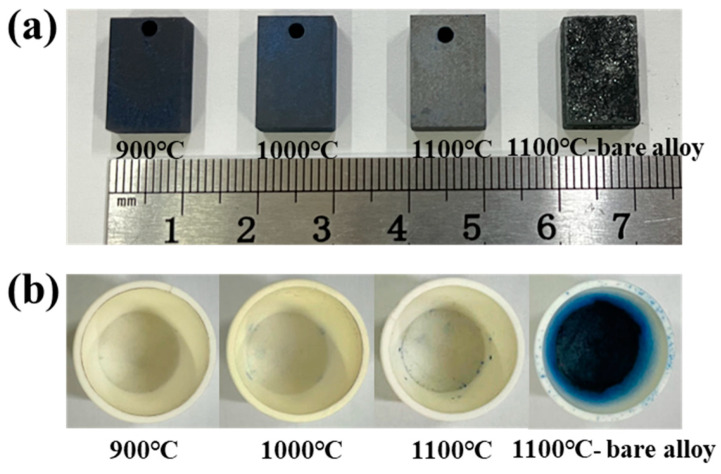
Appearances of (**a**) the coating, bare alloy, and (**b**) crucibles after oxidation for 100 h.

Figure 8a shows the appearance of the coating and bare alloy after oxidation for 100 h at 900–1100 °C. The surface morphologies of the three coating samples are obviously smoother compared to that of the bare alloy sample, indicating superior oxidation resistance of the aluminide coating. It can be seen that the coating surfaces after oxidation exhibit the characteristic of blue color for all three coating samples, which becomes lighter as the temperature increases. In the bare alloy sample, it can be seen from Figure 8a that the surface is relatively rough with obvious flaking after oxidation at 1100 °C, indicating a severe oxide scale spallation on the bare alloy surface. Figure 8b shows the appearance of the crucibles of the coating and bare alloy after oxidation for 100 h. The crucibles of three coating samples are relatively clean compared to that of the bare alloy sample, although some oxides can also be observed in the crucible of the coating sample after oxidation for 100 h at 1100 °C. The crucible inner walls of the bare alloy sample become blue after oxidation for 100 h at 1100 °C with oxide flakes all over the walls. Compared with the rough surface of the bare alloy sample in Figure 8a, these oxide flakes may be the result of the oxide scales spallation after oxidation for 100 h at 1100 °C.

**Figure 9:**
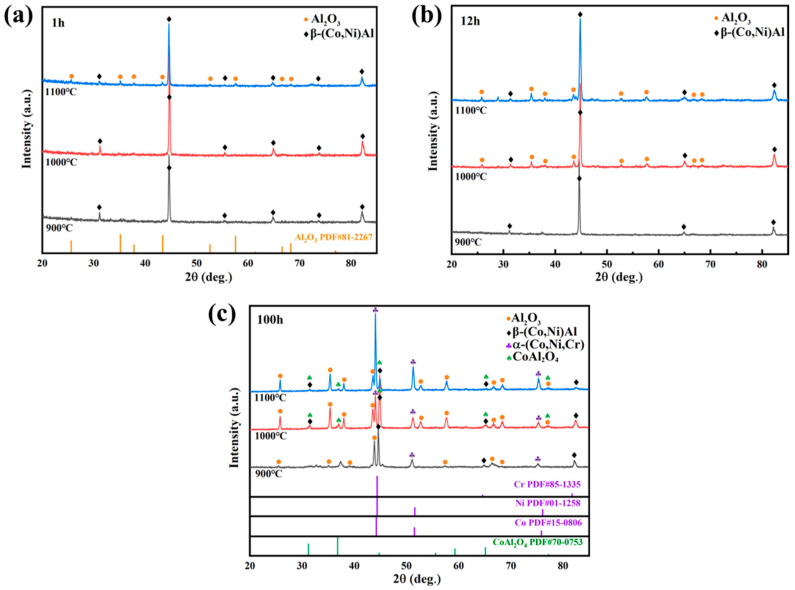
XRD patterns of the coating after oxidation for (**a**) 1 h, (**b**) 12 h, and (**c**) 100 h at different temperatures.

The XRD patterns of the coating after oxidation at 900, 1000, and 1100 °C for 1, 12, and 100 h are shown in Figure 9. It can be seen that the oxidation product of the coating at different temperatures is mainly Al_2_O_3_, some minor oxides like CoAl_2_O_4_ can also be detected after oxidation for 100 h at 1000 and 1100 °C. However, the onset of Al_2_O_3_ formation varies with temperature, with higher oxidation temperatures leading to its earlier and more abundant formation. With the increase in oxidation temperature and oxidation time, the intensity of the diffraction peak of Al_2_O_3_ is higher, which indicates that the Al_2_O_3_ scale is thicker. The diffraction peaks of α-(Co,Ni,Cr) (abbreviated as α in the following paragraph) appear after oxidation for 100 h at 900, 1000, and 1100 °C.

### 3.4. The Microstructure of the Coatings After Oxidation

**Figure 10:**
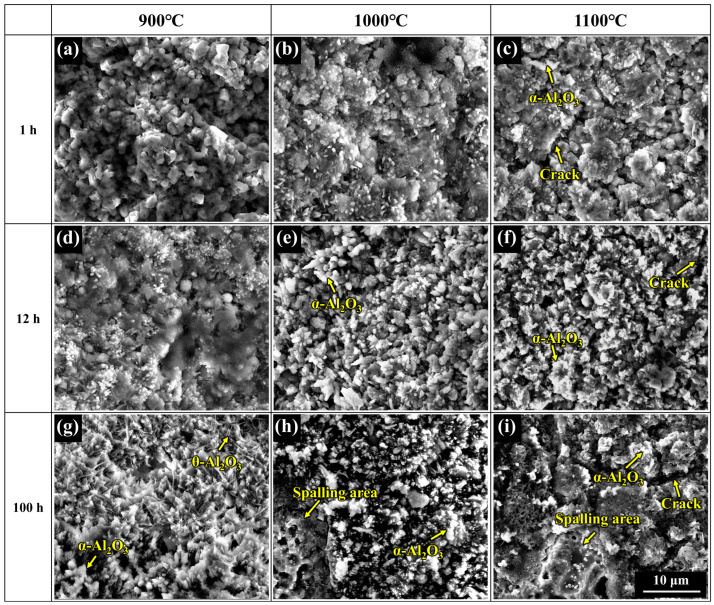
Surface morphologies of the coating after oxidation at (**a**,**d**,**g**) 900 °C, (**b**,**e**,**h**) 1000 °C, (**c**,**f**,**i**) 1100 °C for (**a**–**c**) 1 h, (**d**–**f**) 12 h, and (**g**–**i**) 100 h.

Figure 10 shows the surface morphology of the coatings after oxidation at 900–1100 °C for 1, 12, and 100 h. After oxidation at 900 °C for 1 h, the particles on the surface of the coating became rounded compared to that before oxidation (Figure 4b). The pores between particles became smaller, and oxides were initially formed, which can be seen in Figure 10a. After oxidation at 900 °C for 12 h, some small particles could be observed on the surface, which were characterized by white particles with extremely small size dispersing on the surface (see Figure 10d). After oxidation at 900 °C for 100 h, the white particles were prevailing on the surface, which could be determined to be Al_2_O_3_ by XRD results (Figure 9c). In addition, the shape of the Al_2_O_3_ oxide showed two characteristics: needle-like and lumpy. The needle-like and lumpy shape of Al_2_O_3_ oxide can be inferred to be θ-Al_2_O_3_ and α-Al_2_O_3_, respectively, based on the shape characteristic. As the oxidation temperature increases to 1000 °C, the white particles are more obvious, which can be observed in Figure 10b. After oxidation at 1000 °C for 12 h, the oxide scales were covering the coating surface, indicating significant oxidation on the coating. The oxidation on the coating at 1000 °C was becoming more severe after oxidation for 100 h with oxide scales spallation occurring, as shown in Figure 10h. At 1100 °C, the α-Al_2_O_3_ has already been formed on the surface of the coating after 1 h of oxidation, and cracks could also be observed in Figure 10c. After oxidation for 100 h at 1100 °C, large spalling areas appeared on the surface, accompanying the increasing number and depth of cracks.

**Figure 11:**
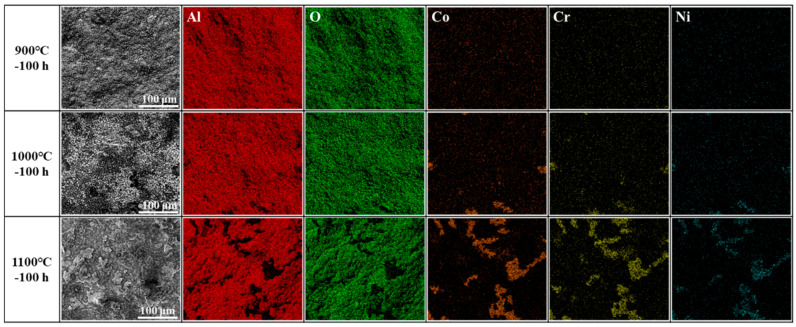
Elemental distribution maps of surface of the coating after oxidation at different temperatures for 100 h.

Figure 11 shows the elemental distribution maps of the coating surface after oxidation at 900–1100 °C for 100 h. It can be found that the element distributions on coating surface after oxidation at 900 °C for 100 h were relatively uniform with enrichment of Al and O on the surface. With oxidation temperature increasing to 1000 °C, the main elements on the surface were still Al and O. But, the enrichment of Co, Cr, and Ni in the oxide scale spallation areas could also be observed. The element distributions after oxidation at 1100 °C were similar with those at 1000 °C. But, the oxide scale spallation areas were larger compared to that at 1000 °C, resulting in a larger area of Co, Cr, and Ni enrichment.

**Figure 12:**
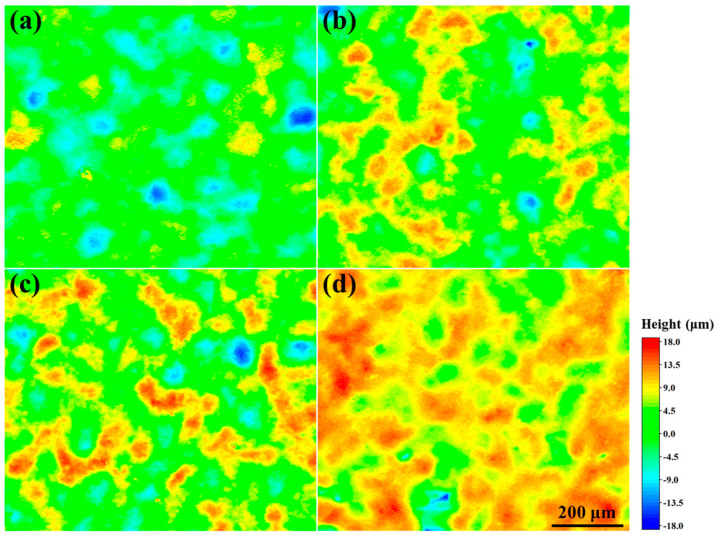
Laser spectroscopy confocal microscope profiles of the coating (**a**) before oxidation and after oxidation at temperatures of (**b**) 900, (**c**) 1000, and (**d**) 1100 °C for 1 h.

The height profiles of the surface before and after oxidization at 900–1100 °C for 1 h are shown in Figure 12. It can be seen that the coating has a relatively uniform surface with a small surface height variation before oxidation. After oxidation, the height of some areas of the surface increases due to the oxide generation on the coating surface. As the oxidation temperature increases, the surface of the coating becomes more and more uniform. This is due to the transformation of the coating from local oxidation to uniform oxidation [30].

**Figure 13:**
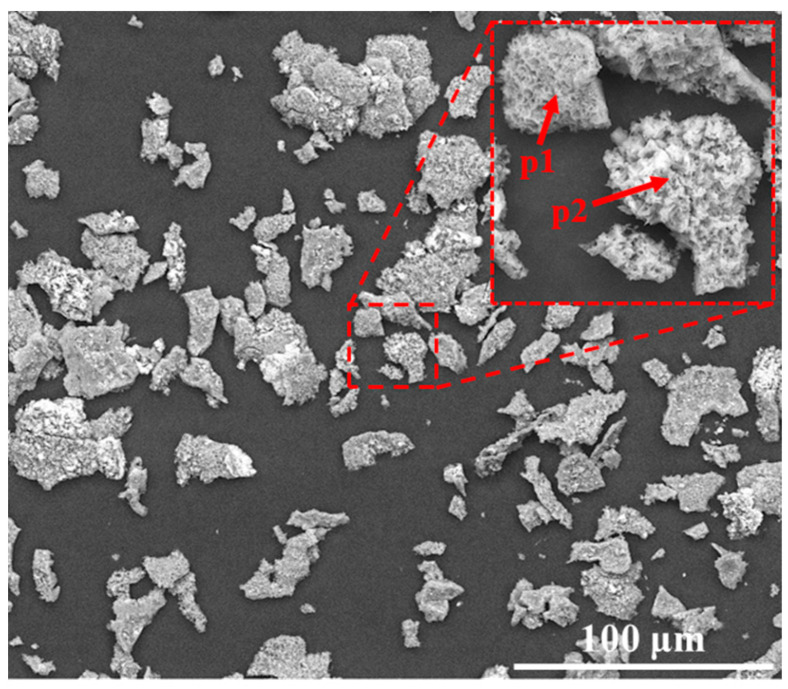
Morphology of oxide residue in the crucible of the coating at 1100 °C for 100 h.

The residual oxide flakes in the crucible of the coating after oxidation at 1100 °C for 100 h was characterized by SEM and EDS, as shown in Figure 13. It can be found that the morphology of the residual oxide is similar to the surface morphology of the coating after oxidation (see Figure 10i). The composition of the oxide flakes in the crucible was detected by EDS, as shown in Table 2. According to the composition analysis of p1 and p2, the residual oxide in the crucible was Al_2_O_3_.

**Figure 14:**
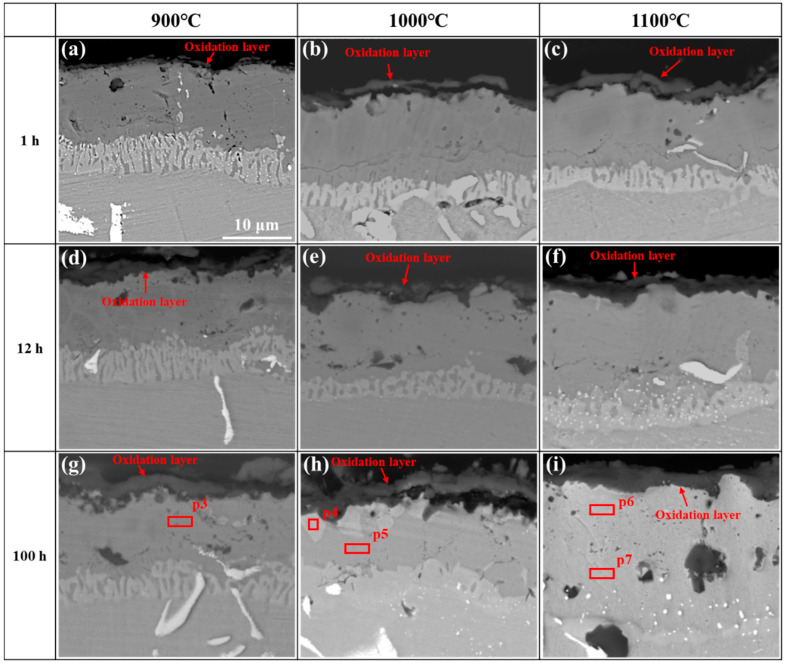
Cross-sectional morphologies of the coating after oxidation at (**a**,**d**,**g**) 900 °C, (**b**,**e**,**h**) 1000 °C, (**c**,**f**,**i**) 1100 °C for (**a**–**c**) 1 h, (**d**–**f**) 12 h, and (**g**–**i**) 100 h.

Figure 14 exhibits BSE images of the cross-section morphologies of the coating after oxidation at 900–1100 °C for 1, 12, and 100 h. It can be seen from Figure 14a that a relatively loose oxide layer is formed on the surface of the coating, and the oxide layer is discontinuous after oxidation at 900 °C for 1 h. With the oxidation time increasing, the oxide layer gradually becomes dense and continuous. With the temperature increasing, the oxidation of the coating was becoming significant. It can be seen from Figure 14c that a continuous oxide layer has been formed after oxidation at 1100 °C for 1 h. This is consistent with the surface morphologies of the coating after oxidation, which was shown in Figure 10c. After the oxidation of 1100 °C for 100 h, the thickness of oxide layer can be estimated to be ~3 μm (see Figure 14i).

In the diffusion layer, it is seen from Figure 14h that the Cr-rich diffusion layer almost completely disappears after oxidation at 1000 °C for 100 h. This was similar to the results found by Fan et al. [31] in their study on the oxidation of Co-modified aluminide coatings. A bright phase (p4) and dark phase (p5) were found in the outer layer of the coating after oxidation at 1000 °C for 100 h. According to the EDS spectrum results (Table 2), the bright phase is rich in Co, Cr, and Ni, with a relatively low content of Al, which can be determined to be the α phase combined with the XRD results (Figure 9c). This indicates that Cr atoms were diffusing from the diffusion layer to the outer layer of the coating during oxidation. The dark phase (p5) contained a relatively high content of Al, which can be determined to be the β phase. When the coating was oxidized at 1100 °C for 12 h (Figure 14f), it was found that the Cr-rich diffusion layer obviously faded, indicating that the diffusion of the Cr element is significant at 1100 °C. After oxidation at 1100 °C for 100 h, it was also found that the diffusion layer of Cr-rich completely disappeared, and there was no obvious second phase in the outer layer of the coating (see Figure 14i). In the XRD pattern of Figure 9c, it can also be seen that the diffraction peaks of the β phase become very weak, indicating that the content of the β phase in the outer layer is extremely low. The chemical compositions of p6 and p7 were analyzed (see Table 2), and it was found that the chemical compositions of the two locations were almost the same, showing that the coating was relatively uniform at this time.

**Figure 15:**
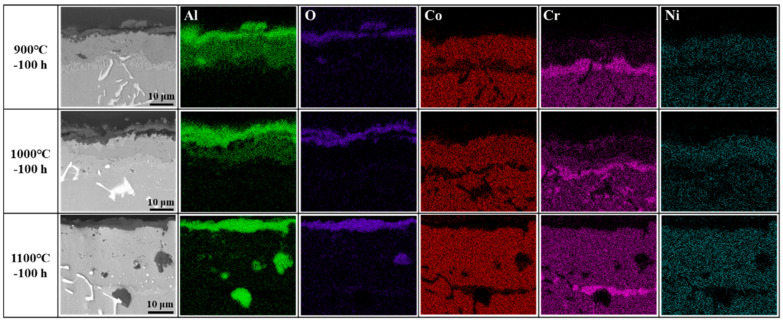
Elemental distribution maps of cross-section of the coating after oxidation at different temperatures for 100 h.

Figure 15 demonstrates the elemental distribution maps of the coating cross-section after oxidation at 900–1100 °C for 100 h. It can be observed that with the increase in oxidation temperature, Al gradually diffuses outward to combine with O to form oxide scales, while Cr gradually diffuses from the diffusion layer of the coating to the outer layer. The diffusion layer gradually disappeared, and the composition of the outer layer of the coating was gradually consistent.

## 4. Discussion

From the above results, it can be concluded that the aluminide coating prepared in this study on K6509 alloy is a double-layer structure with an outer layer and diffusion layer. The oxidation behavior of this coating at 900, 1000, and 1100 °C shows significant differences.

### 4.1. Mechanism of the Coating Formation

It is clear that the aluminide coating prepared in this study on K6509 alloy is composed of a β-(Co,Ni)Al phase outer layer and a Cr-rich phase diffusion layer. The surface of the coating is composed of particles with different sizes and irregular shapes. This is similar to the NiAl coating prepared by Gao and Zou [32], but the difference is that the particles on the surface of the coating prepared by them are smoother. There are many small pores on the surface of the coating as seen in Figure 4b, but in combination with the cross-sectional images of the coating in Figure 3a, it can be shown that there are no pores in the interior of the coating. This indicates that the coating is dense, and these holes appear only near the surface of the coating.

The principle of vapor phase aluminizing is similar to that of pack cementation [33]. In the presence of NH_4_Cl activator, active Al atoms are generated. The active Al atoms migrate towards the surface of the substrate and are deposited. Finally, the active Al atoms diffuse with the Co and Ni elements in the substrate to produce an aluminide coating. In this study, the following reactions may occur during vapor phase aluminizing [34]:

(2) $$NH_{4}Cl\to NH_{3}+HCl$$

(3) $$3HCl+Al\to Al{Cl}_{3}+3/2H_{2}$$

(4) $$Al{Cl}_{3}+Al\to Al{Cl}_{2}+AlCl$$

(5) $$3AlCl\to 2\left[Al\right]+Al{Cl}_{3}$$

(6) $$3Al{Cl}_{2}\to \left[Al\right]+2Al{Cl}_{3}$$

(7) $$\left[Al\right]+\alpha -\left(Co,Ni\right)\to \beta -\left(Co,Ni\right)Al$$

**Figure 16:**
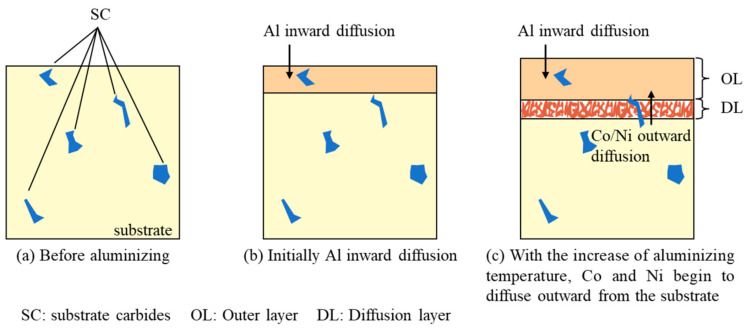
Schematic drawing of the formation mechanism of the coating.

There are two ways to grow aluminide coatings [35]: inward growth and outward growth. The aluminide coating prepared in this study exhibited an inward growth pattern. Figure 16 shows the schematic drawing of the mechanism of the coating formation. During the initial growth of the coating, Al diffuses from the surface of the substrate to the interior first because Al diffuses faster than Co and Ni in (Co,Ni)Al [9,36]. So, the carbides of the substrate will remain in the coating [37], as shown in Figure 3a. With the increase in the aluminizing temperature, the diffusion rate of Co and Ni increases [38,39], and Co and Ni diffuse from the substrate interior to the surface. Therefore, the content of Co and Ni decreases, and the content of Al increases in the region of the substrate near the coating, forming a diffusion zone. Due to the low solubility of Cr in the β phase, it precipitates in the diffusion layer in the form of precipitated phase [9]. As shown in Figure 4a, the surface morphology of the coating appears relatively rough. This is because, during the vapor phase aluminizing process, carbides in the substrate act as localized barriers, disrupting the uniform diffusion of Al, Co, and Ni, which also affects the growth of aluminides. The uneven growth of aluminides results in the rough surface morphology of the coating [40].

### 4.2. Oxidation Behavior of the Coating

From the above results, the aluminide coating prepared on K6509 alloy shows good oxidation resistance at 900–1100 °C. The coating forms a uniform and dense oxide layer after oxidation to prevent the substrate from oxidation. During prolonged oxidation at high temperatures, the aluminide coating does not spall from the substrate, but oxide-layer spallation could be observed (see Figure 10h,i). Oxide-layer spallation was caused by the mechanical stresses developed during oxide growth and the thermal expansion coefficient mismatch between the oxide and the substrate [41].

**Figure 17:**
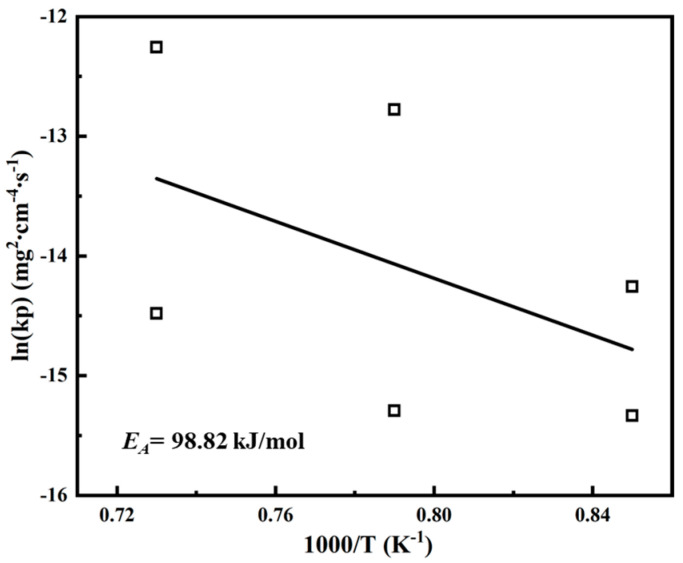
Arrhenius plot for the oxidation of the coating at 900–1100 °C.

As shown in Figure 6a, the oxidation of the coating at 900–1100 °C all obeys the parabolic law. Compared to the previously studied Co-Al alloys [42], the oxidation rate constant of the coating at 1000 °C is comparable to that of Co-18Al ($K_{P}$ = 87 × 10^−7^ mg^2^·cm^−4^·s^−1^). The relationship between the oxidation rate constant $K_{P}$ and the oxidation temperature can be expressed by the Arrhenius equation:

(8) $$k_{p}=k_{0}exp\left(-\frac{E_{A}}{RT}\right)$$

where $k_{0}$ is a constant, $E_{A}$ is the oxidation activation energy, R is the gas constant, and T is the temperature. The oxidation activation energy $E_{A}$ of the coating can be calculated through the liner fitting of the ln($K_{P}$) versus the reciprocal of T, as shown in Figure 17. The $E_{A}$ of the coating is 98.82 kJ/mol. The oxidation activation energy of the coating is similar to the activation energy of Al_71_Co_29_ alloy reported in previous studies [41].

It has been reported that the oxidation rate increases with increasing oxidation temperatures [43]. However, it can be found from Figure 6a that the mass gain of the coating at 1000 °C exceeded the mass gain at 1100 °C after 5 h. As shown in Figure 13, the residual oxide flakes in the crucible of the coating after oxidation at 1100 °C for 100 h was Al_2_O_3_ without any volatile oxides, such as Cr_2_O_3_. In addition, there were also no diffraction peaks of volatile substances on the XRD pattern in Figure 9. Hence, it can be inferred that the lower oxidation mass gain at 1100 °C than that at 1000 °C was not caused by the generation of volatile substances.

At the initial stage of oxidation, the coating was exposed to the air directly, so the oxidation rate at this stage is fast, and the oxidation mass gain increases rapidly [20]. With the prolongation of oxidation time, the Al_2_O_3_ layer becomes continuous and dense, leading to a decreased interfacial reaction between oxygen and the coating and the decrease in the oxidation rate. Aluminum ions need to diffuse outward through the Al_2_O_3_ layer and oxygen ions diffuse inward through the Al_2_O_3_ layer [44,45,46] to complete the reaction. The continuous and dense Al_2_O_3_ layer put a great obstruction to the diffusion of aluminum ions and oxygen ions, resulting in a reduction in the oxidation rate and oxidation mass gain. The earliest transition from the initial to later stage may be ascribed to the faster formation of dense Al_2_O_3_ layer on coating, which effectively separates the coating and air. It can be seen from Figure 6b that the three curves all exhibit two stages with a higher oxidation rate in the initial stage and lower oxidation rate in the later stage. The transition points between two stages are different for different oxidation temperatures, which are 24, 24, and 3 h for 900, 1000, and 1100 °C, respectively. This may be ascribed to the faster formation of the dense Al_2_O_3_ layer at 1100 °C, which can be verified from Figure 14 b,c, which shows that the Al_2_O_3_ layer generated on the coating at 1100 °C is denser than at 1000 °C for 1 h. As the oxidation proceeds, the Al_2_O_3_ layer generated by the coating at 1100 °C becomes more continuous and denser. This may be the reason why the mass gain of the coating after 5 h at 1100 °C is less than at 1000 °C.

The θ-Al_2_O_3_ produced by the oxidation of the coating at 900 °C is a metastable structure with a fast growth rate and loose structure [47,48]. In contrast, α-Al_2_O_3_ produced at 1000 and 1100 °C is a corundum structure with a slow growth rate, high stability, and dense structure [49]. The temperature at which θ-Al_2_O_3_ transforms to α-Al_2_O_3_ is 1000 °C [20], so the coating may first become θ-Al_2_O_3_ and then transform to α-Al_2_O_3_ when oxidation occurs at 1000 °C. Due to the fact that θ-Al_2_O_3_ is looser structure than α-Al_2_O_3_, it results in the coating generating the oxide layer at 1000 °C which is not as dense as the one at 1100 °C. 

**Figure 18:**
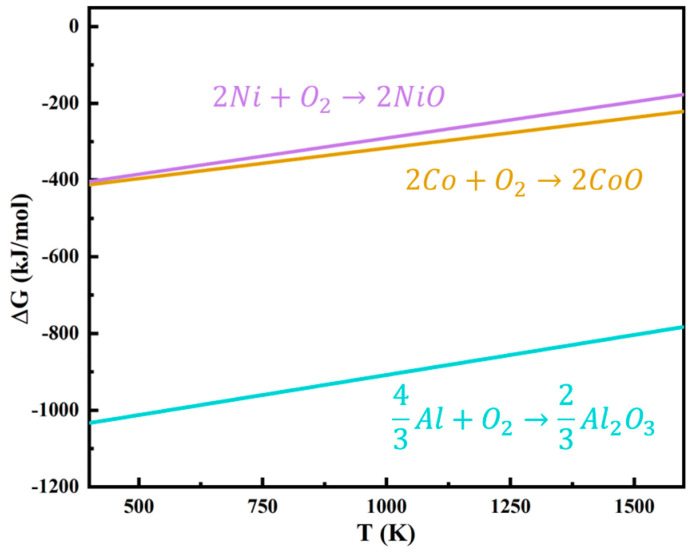
Gibbs free energies of metal oxidation reactions at different temperatures, redrawn from [41].

Figure 18 illustrates the Gibbs free energy of Al_2_O_3_, CoO, and NiO at different temperatures. It can be found that at the same temperature, Al_2_O_3_ has the smallest Gibbs free energy, followed by CoO, and the largest is NiO. Spinel CoAl_2_O_4_ were formed after oxidation of the coating at 1000 and 1100 °C for 100 h, as illustrated in Figure 9c. This is because the Gibbs free energy from the generation of CoO is lower than that of NiO, and the growth rate of CoO is greater than that of NiO [50], so Co is easily oxidized and forms CoO. CoO reacts with Al_2_O_3_ to produce CoAl_2_O_4_, as shown in the following expression [51]:

(9) $$CoO+{Al}_{2}O_{3}\to Co{Al}_{2}O_{4}$$

From Figure 15 and Table 2, it can be seen that as the oxidation temperature increases, more Al in the coating is consumed, resulting in a larger aluminum-poor zone in the coating. During oxidation, the Al atoms near the surface of the coating is consumed first, and then the Al atoms inside the coating diffuses to the surface under the driving force of the chemical composition gradient. The outward diffusion of Al decreases the Al content in the coating (e.g., p4 in Figure 14h), leading to the transformation of the β phase to the α phase. The transformation of β phase into α phase can be evidenced in the Al-Co phase diagram [52]. Since the solubility of Cr in α phase is higher than β phase [53], the Cr-rich diffusion layer is gradually dissolved into the α phase accompanying with Cr diffusion to the outer layer of the coating. The contents of p6 and p7 in Figure 14i are almost the same (see Table 2), indicating that the composition of the coating becomes uniform after oxidation at 1100 °C for 100 h, at which the coating is almost completely converted to the α phase. The possible phase transformations of the coating during the oxidation are as follows:

(10) β→β+α→α

**Figure 19:**
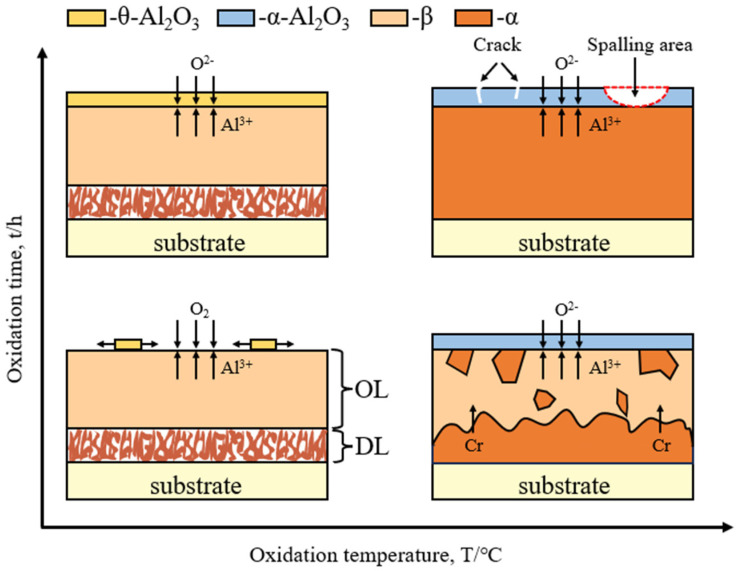
Schematic drawing of the oxidation behavior of the coating at different temperatures and different times.

From the above results, it can be seen that the oxidation resistance of the coating is related to the type of Al_2_O_3_ formed, interfacial reaction, diffusion reaction, dissolution of the diffusion layer, transformation of phases, and spalling of the oxide layer as illustrated in Figure 19.

## 5. Conclusions

In this study, an aluminide coating was successfully prepared on the cobalt-based superalloy through a vapor phase aluminizing process, which is composed of an outer layer of β-(Co,Ni)Al phase and a diffusion layer of Cr-rich phase. The following conclusions can be drawn:

1. The mechanism of the aluminide coating formation on the cobalt-based superalloy in this study is dominated by the inward diffusion of Al.
2. The coating after oxidation generates an oxide layer containing mainly Al_2_O_3_. As the oxidation temperature increases and the oxidation time prolongs, the oxide layer flakes and cracks. The oxidation of the coating at 900 °C produces metastable needle-like θ-Al_2_O_3_ and at 1000 and 1100 °C produces stable lumpy α-Al_2_O_3_.
3. The reason that the weight gain of the coating oxidation at 1100 °C for 5 h is less than that at 1000 °C may be due to the rapid generation of a dense oxide layer of the coating at 1100 °C, which slows down the diffusion of Al and O ions and reduces the oxidation rate.
4. In high-temperature oxidation, Al in the coating is consumed in large quantities. Hence, the β phase in the coating gradually converts into the α phase, leading to the dissolution of the diffusion layer.

## Figures and Tables

**Table 1:** The compositions of Co-based superalloy K6509 (wt%).

Elements	Cr	Ni	W	Ta	C	Ti	Zr	Si	Co
compositions	24.7	9.9	6.5	3.5	0.7	0.2	0.4	0.2	53.9

**Table 2:** EDS point analysis results (at.%) at p1, p2, p3, p4, p5, p6, and p7 for the sample shown in Figure 12 and Figure 13.

Elements	p1	p2	p3	p4	p5	p6	p7
Al	58.3	49.5	39.7	5.3	23.1	4.2	3.5
O	40.8	48.9	-	-	-	-	-
Co	0.4	1.2	45.4	62.0	49.2	57.4	57.6
Cr	0.4	0.3	5.6	22.0	9.8	23.7	24.1
Ni	0.1	0.1	9.3	9.2	17.0	13.1	12.8
W	-	-	-	1.5	0.9	1.6	2.0

## Data Availability

The original contributions presented in the study are included in the article, further inquiries can be directed to the corresponding authors.
